# *In vivo* Reconstitution of Algal Triacylglycerol Production in *Saccharomyces cerevisiae*

**DOI:** 10.3389/fmicb.2016.00070

**Published:** 2016-02-15

**Authors:** Chun-Hsien Hung, Kazue Kanehara, Yuki Nakamura

**Affiliations:** ^1^Institute of Plant and Microbial Biology, Academia SinicaTaipei, Taiwan; ^2^Precursory Research for Embryonic Science and Technology (PRESTO), Japan Science and Technology AgencySaitama, Japan

**Keywords:** triacylglycerol, *Chlamydomonas reinhardtii*, *Saccharomyces cerevisiae*, diacylglycerol acyltransferase type 2, metabolic engineering

## Abstract

The current fascination with algal biofuel production stems from a high lipid biosynthetic capacity and little conflict with land plant cultivation. However, the mechanisms which enable algae to accumulate massive oil remain elusive. An enzyme for triacylglycerol (TAG) biosynthesis in *Chlamydomonas reinhardtii*, CrDGTT2, can produce a large amount of TAG when expressed in yeast or higher plants, suggesting a unique ability of CrDGTT2 to enhance oil production in a heterologous system. Here, we performed metabolic engineering in *Saccharomyces cerevisiae* by taking advantage of CrDGTT2. We suppressed membrane phospholipid biosynthesis at the log phase by mutating *OPI3*, enhanced TAG biosynthetic pathway at the stationary phase by overexpressing *PAH1* and *CrDGTT2*, and suppressed TAG hydrolysis on growth resumption from the stationary phase by knocking out *DGK1*. The resulting engineered yeast cells accumulated about 70-fold of TAG compared with wild type cells. Moreover, TAG production was sustainable. Our results demonstrated the enhanced and sustainable TAG production in the yeast synthetic platform.

## Introduction

Biofuel production is highly demanded as an alternative to the limited fossil fuels. The current fascination with eukaryotic algae as a resource of biofuel production stems from a high lipid biosynthetic capacity and little competition for land required for plant cultivation. However, technical difficulty in gene manipulation of many eukaryotic algae species hampers potential usefulness of these organisms as a synthetic platform of metabolic engineering to produce biofuel. On the other hand, *Saccharomyces cerevisiae* is an advanced model unicellular eukaryotic microorganism for metabolic engineering as well as basic molecular biological studies on metabolism.

**Figure 1:**
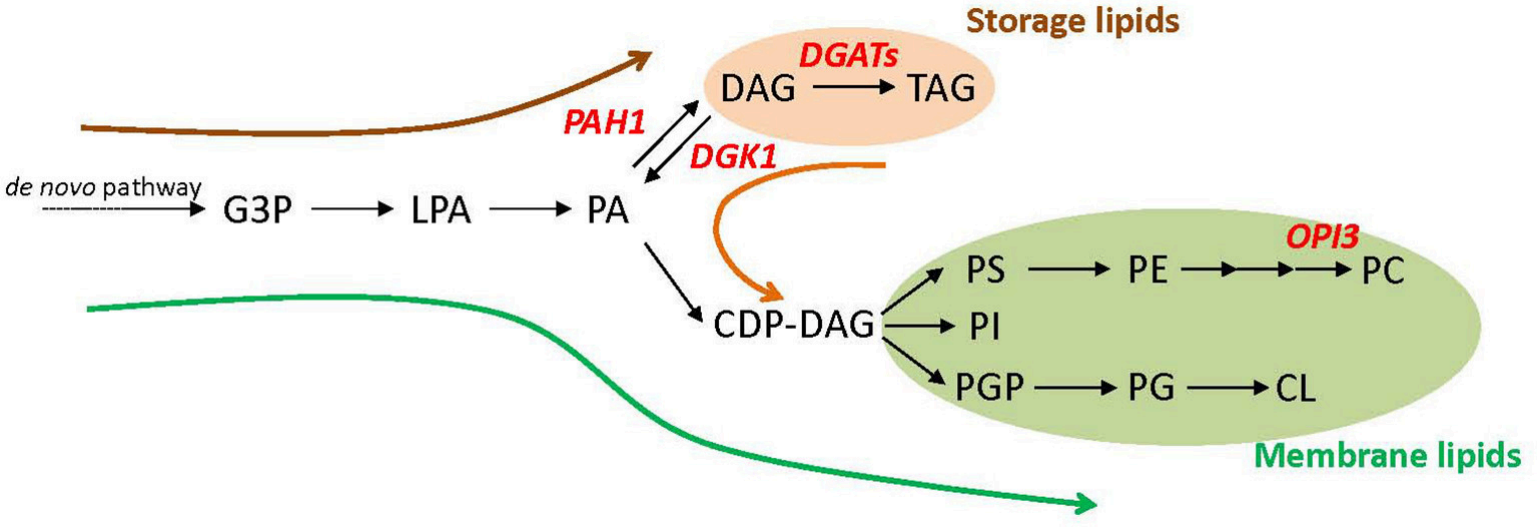
**Schematic representation of glycerolipid metabolism in *Saccharomyces cerevisiae***. Dominant metabolic flux at three different growth phases (log phase, green arrow; stationary phase, brown arrow; growth resumption from the stationary phase, orange arrow) and key metabolic genes engineered in this study (in red letters) are shown. CDP-DAG, cytidine diphosphate-diacylglycerol; CL, cardiolipin; DAG, *sn*-1,2-diacylglycerol; G3P, glycerol 3-phosphate; LPA lysophosphatidic acid; PA, phosphatidic acid; PC, phosphatidylcholine; PE, phosphatidylethanolamine; PG, phosphatidylglycerol; PGP, phosphatidylglycerophosphate; PI, phosphatidylinositol; PS, phosphatidylserine; TAG, triacylglycerol.

Triacylglycerol (TAG) is a major source of biodiesel (Durrett et al., 2008; Hu et al., 2008), which is synthesized from *sn*-1,2-diacylglycerol (DAG) by the catalysis of acyltransferase in many organisms such as *S. cerevisiae, Chlamydomonas reinhardtii*, and *Arabidopsis thaliana* (Sandager et al., 2002; Zhang et al., 2009; Hung et al., 2013). Because DAG is produced from phosphatidic acid (PA), which is also a substrate for the biosynthesis of major membrane phospholipids, TAG accumulation often competes with membrane lipid biosynthesis (Figure 1). In yeast cells, the primary metabolic flux of glycerolipids differs by the growth status. Biosynthesis of storage lipids such as TAG and membrane lipids such as phospholipids share initial steps of the pathway up to PA production (Henry et al., 2012). In growing cells, newly synthesized glycerolipids are mostly used to produce membrane lipids required for active cell proliferation (Figure 1, green arrow). With entry into the stationary growth phase, the major flux switches to storage lipid biosynthesis. Here, PA is dephosphorylated to DAG by a PA phosphatase encoded by *PAH1* (Han et al., 2006), which is subsequently converted to TAG by diacylglycerol acyltransferases (DGATs; Figure 1, brown arrow). Stationary-phase cells can resume their growth once depleted nutrients are added, such as the dilution of cell culture with fresh medium. This growth resumption activates hydrolysis of accumulated TAG to DAG by a set of TAG lipases (Kurat et al., 2009), and DAG is further phosphorylated by DAG kinase 1 (DGK1) to PA (Han et al., 2008a,b) to provide a substrate for membrane lipid biosynthesis (Figure 1, orange arrow). At this phase, DGK1 encodes a key enzyme involved in phospholipid synthesis and recovery after growth resumption (Fakas et al., 2011a).

*C. reinhardtii* is a model organism for the study of TAG production in the eukaryotic microalgae. DGAT, which represents a major TAG biosynthetic enzyme, catalyzes the transfer of acyl moiety from acyl-CoA to DAG. In *C. reinhardtii*, DGAT is of 2 types: Type 1 with single isogene (*CrDGTT1*) and Type 2 with five isogenes (*CrDGTT1-5*) (Merchant et al., 2012). Interestingly, it was recently reported that heterologous overexpression of *CrDGTT2* in *S. cerevisiae* or *A. thaliana* increased TAG content by 9- or 25-fold, respectively (Hung et al., 2013; Sanjaya et al., 2013), suggesting highly robust enzyme function of CrDGTT2 in a heterologous system.

In this study, we aimed to perform metabolic engineering that achieves enhanced and sustainable TAG production in the synthetic platform of *S. cerevisiae* by taking advantage of *CrDGTT2* as a robust algal gene resource.

## Materials and methods

### Yeast culture conditions

Cells were grown in YPD media (2.0 g of Bacto peptone [Difco 211677], 1.0 g of Bacto yeast extract [Difco 212750], 2.0 g of glucose [Merck 1.08337] in 100 ml of H_2_O produced by Milli-Q [Millipore]) unless otherwise stated. Cells harboring the *URA3* and *LEU2* marker plasmids were grown in synthetic complete media lacking uracil and leucine (SC -Ura, -Leu). The OD_600_ in Figure 3A and Supplementary Figure 1 was measured by diluting fully grown cultures to OD_600_ of 0.1 and cultured for 60 h at 30°C. In Figures 4A–C, yeast cells were freshly inoculated from glycerol stocks. After reaching the stationary phase, the cell culture was diluted with fresh medium to OD_600_ of 0.05 and grown for 60 h to the late stationary phase, then time-course observation was started. The dilution was conducted twice at 60 h intervals. Data are mean±SD from three biological replicates.

### Cloning of plasmid vectors

The yeast expression vector pCH108 was created by replacing the *URA3* marker of pCH078 (Hung et al., 2013) with the *LEU2* marker derived from pRS315. First, XmaI and BclI restriction sites were created by amplifying the 4895-bp fragment of the pCH078 with the primers CH376 and CH377. Next, a 1574-bp fragment of the *LEU2* marker was amplified from pRS315 with the primers CH374 and CH375. The obtained fragments were digested with XmaI and BclI and ligated to construct pCH108. The polymerase chain reaction (PCR) was performed using Phusion polymerase (Finnzymes F-530S, ThermoFisher Scientific) under the following condition; an initial denaturing step of 5 min at 95°C followed by 35 cycles of 95°C for 30 s, 60°C for 30 s, and 72°C for 3 min and then a final extension at 72°C for 5 min. The plasmids and primers are listed in Table 1 and Supplementary Table 1, respectively.

**Table 1:** **List of plasmids used in this study**.

**Plasmid**	**Encoded gene**	**Promoter**	**Vector**	**Source**
pRS313	–	–	pBluescript KS+	ATCC 77142
pRS315	–	–	pBluescript KS+	ATCC 77144
pCH078	–	GPD	pYES2/NTA	Hung et al., 2013
pCH091	*CrDGTT2*	GPD	pYES2/NTA	Hung et al., 2013
pCH108	–	GPD	pYES2/NTA	This work
pCH109	*ScPAH1*	GPD	pYES2/NTA	This work

The 2593-bp open reading frame of *ScPAH1* (YMR165C) was amplified with the primers CH367 and CH368, cloned into pENTR/D-TOPO (Invitrogen, Carlsbad, CA), digested with AscI and NotI, and inserted into AscI/NotI sites of pCH108 to construct pCH109. This plasmid contains GPD promoter for constitutive overexpression (Blazeck et al., 2012).

### Mutant strain construction

To produce CHY034 (*dgk1*Δ::*KANMX, opi3*Δ::*HIS*), a 932-bp fragment of *opi3*Δ::*HIS3* was amplified with the primers CH430 and CH431 from pRS313, and transformed into *dgk1*Δ::*KANMX*. The plasmid pCH109, which overexpresses *ScPAH1* (OE-PAH1), was transformed into CHY034 (*dgk1*Δ::*KANMX, opi3*Δ::*HIS*) to produce CHY138 (Δ*dgk1*Δ*opi3* OE-PAH1). Then, the plasmid pCH091, which overexpresses *CrDGTT2* (OE-CrDGTT2), was transformed into CHY138 to produce CHY101, CHY140, CHY141, and CHY142 (Δ*dgk1*Δ*opi3* OE-PAH1 OE-CrDGTT2). The transformation was performed as described previously by mixing the plasmid with the PLATE reagent, salmon sperm DNA and yeast cells (Gietz and Woods, 2002). The yeast strains are listed in Table 2.

**Table 2:** **List of *S. cerevisiae* strains used in this study**.

**Strain**	**Genotype**	**Source**
BY4741	*MATa, his3Δ0 leu2Δ0 met15Δ0 ura3Δ0*	–
Δ*dgk1*	*dgk1*Δ::*KANMX*, BY4741	Thermo Scientific
CHY034	*dgk1*Δ::*KANMX, opi3*Δ::*HIS*, BY4741	This work
CHY044	pCH078, pCH108, BY4741	This work
CHY101	pCH091, pCH109, *dgk1*Δ::*KANMX, opi3*Δ::*HIS*, BY4741	This work
CHY138	pCH109, *dgk1*Δ::*KANMX, opi3*Δ::*HIS*, BY4741	This work
CHY140	pCH091, pCH109, *dgk1*Δ::*KANMX, opi3*Δ::*HIS*, BY4741	This work
CHY141	pCH091, pCH109, *dgk1*Δ::*KANMX, opi3*Δ::*HIS*, BY4741	This work
CHY142	pCH091, pCH109, *dgk1*Δ::*KANMX, opi3*Δ::*HIS*, BY4741	This work

### Lipid analysis

Lipid analysis was performed as follows using yeast cells obtained from 100 ml of culture by centrifugation (3000 × g for 5 min). Cells were lyophilized using freeze drier (Alpha 1-2, Martin Christ Gefriertrocknungsanlagen GmbH, Germany) and dry cell weight was measured with fine weighing scale (BEL engineering, Cat. No. M214Ai, Italy). Prior to the lipid extraction, cells were treated with pre-heated isopropanol containing 0.01% butylated hydroxytoluene at 75°C for 15 min to inactivate phospholipase activity. Total lipids were extracted essentially as described previously using chloroform-methanol solvent system (Folch et al., 1957) with the ratio of solvent (by vol): sample (by weight) as 20:1 for 100 mg of sample. Briefly, cells were suspended in chloroform:methanol (1:2, by vol) to homogenity, followed by addition of chloroform and deionized water (dH_2_O). Cell suspension was vortexed vigorously and left for 30 min at room temperature. Organic phase was recovered and residual lipids in cell pellet were extracted twice by vigorous vortex with chloroform. The combined organic extracts were vortexed with 1% KCl solution, and separated organic phase was washed once with dH_2_O. Lipids were dried up under nitrogen stream, dissolved in chloroform:methanol (2:1, by vol) and stored at −30°C till use. Each lipid class was separated by thin layer chromatography (Silica Gel 60 F254 plate, Merck). Separation of TAG was as described previously (Hung et al., 2013) and of phospholipids involved two-dimensional separation with the solvent system of chloroform/methanol/ammonia (65:35:5, by vol) for the first dimension and chloroform/acetone/methanol/acetic acid (50:20:10:10, by vol) for the second dimension based on the previous report (Nelson, 1967). Lipid spots were identified by spraying primuline solution, scraped off and acyl moieties hydrolyzed and methylesterified to fatty acid methyl esters (FAMEs) with HCl-methanol solution by incubating the samples at 85°C for 2 h including pentadecanoic acid (15:0) as internal standard. After the incubation, the FAMEs were extracted with hexane, dried up under the nitrogen gas stream, eluted in 50 μl of hexane, and quantified with gas chromatography (GC-2010; Shimadzu, Kyoto, Japan) with FID detector (FID-2010 Plus; Shimadzu, Kyoto, Japan) equipped with a ULBON HR-SS-10 column (Shinwa Chemical Industries, Japan) (Nakamura et al., 2003). Amount of TAG was shown in % (w/w) of dry cell weight. Composition of fatty acid and phospholipid classes were shown by mol% based on the FAMEs quantified with gas chromatography. Data were averaged by three biological replicates with standard deviations as error bars.

### Nile red staining and microscopy of lipid droplets

Nile red staining and observation of lipid droplets were as described previously (Hung et al., 2013) using Nile red (Fluka, Cat. No. 72485) and DeltaVision system (Applied Precision) with a 100x objective lens (NA = 1.4) and a CoolSNAP HQ CCD camera (Photomotrics) controlled by softWoRx Suite (Applied Precision). Normarski differential interference contrast (DIC) microscopy image was taken as previously described (Fu et al., 2010).

### Transmission electron microscopy

Yeast cells were grown to the stationary phase in synthetic complete medium lacking uracil and leucine (SC -Ura, -Leu) containing 8% glucose at 30°C for 48 h. Samples were frozen in a high-pressure freezer (Leica EM PACT2) at 2000–2050 bar. Freeze-substitution was performed in anhydrous ethanol (containing 0.2% glutaraldehyde and 0.1% uranyl acetate) with an automatic freeze substitution system (Leica EM AFS2). The samples were first kept at −85°C for 3 days, then switched to −60°C, −20°C, 0°C, and room temperature at 1-day intervals. After 2 times of rinse with ethanol for 12 h, the samples were embedded by infiltrating LR White resin. Ultrathin sections (70–90 nm) were prepared with use of Reichert Ultracut S or Lecia EM UC7 (Leica, Vienna, Austria) and collected on 100-mesh copper grids. The sections were stained with 5% uranyl acetate in 50% methanol for 10 min and 0.4% lead citrate for 4–6 min. Sections were observed under a transmission electron microscope (Philips CM 100) at 80 KV and images were taken with use of a Gatan Orius CCD camera.

## Results

### Metabolic engineering of *S. cerevisiae* to enhance TAG production

**Figure 2:**
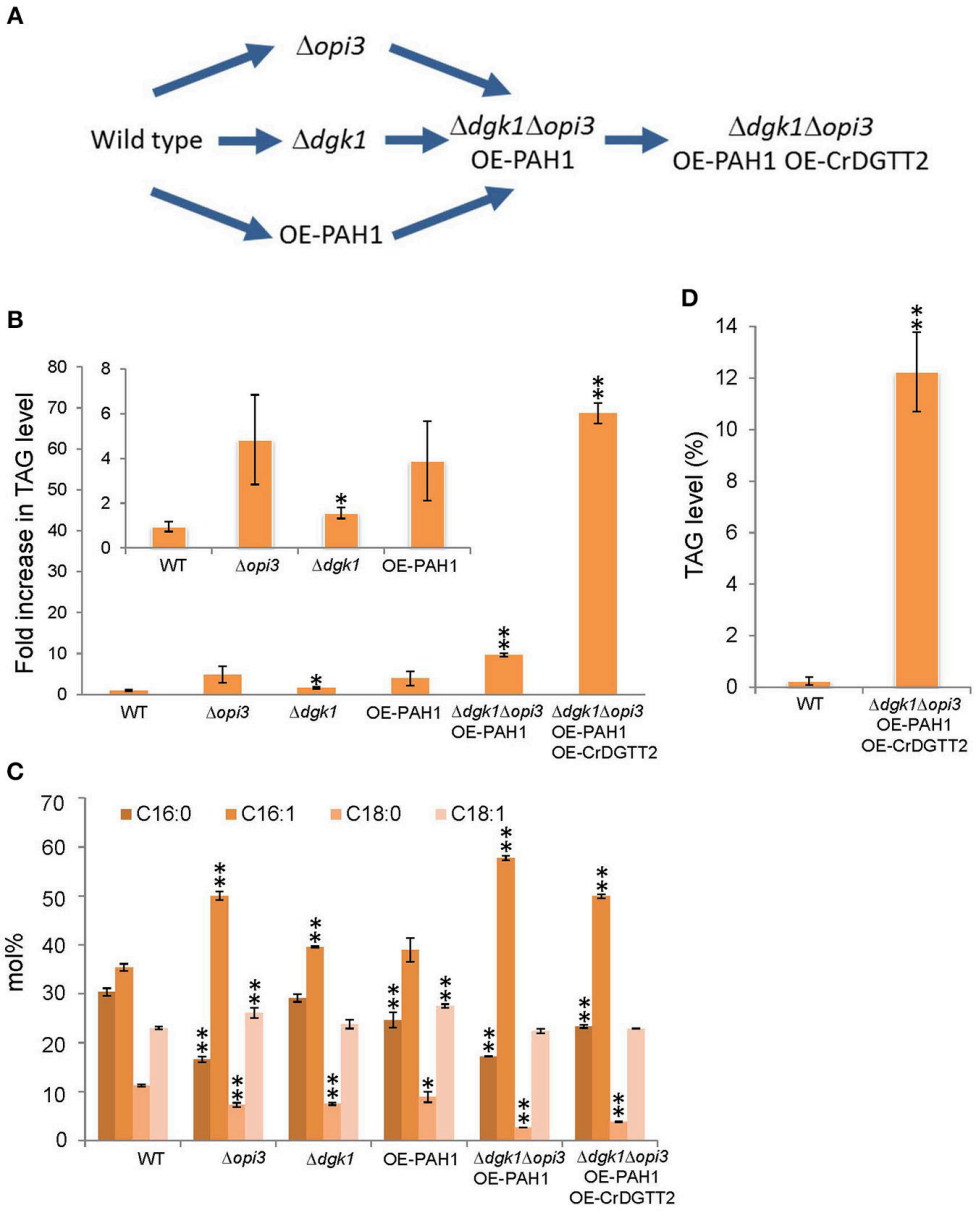
**Procedures for metabolic engineering to enhance TAG production in *S. cerevisiae*. (A)** Schematic representation of the procedures. **(B)** TAG levels of early stationary phase were quantified in the strains of wild type (WT), Δ*opi3*, Δ*dgk1*, OE-PAH1, Δ*dgk1*Δ*opi3* OE-PAH1, and Δ*dgk1*Δ*opi3* OE-PAH1 OE-CrDGTT2 shown as fold increase compared to the wild type strain. **(C)** Fatty acid composition (mol%) of TAG analyzed in **(B)**. **(D)** TAG production of Δ*dgk1*Δ*opi3* OE-PAH1 OE-CrDGTT2 strain at stationary phase by dry cell weight basis. Data are mean ± SD from three biological replicates. Asterisks indicate statistical significance by Student's *t*-test (^*^P < 0.05; ^**^P < 0.01). WT, wild type; OE, overexpression; 16:0, palmitic acid; 16:1, palmitoleic acid; 18:0, stearic acid; 18:1, oleic acid.

To enhance TAG levels by metabolic engineering, we genetically manipulated *S. cerevisiae* by considering the following 3 points: (1) suppressing metabolic flux to membrane lipid biosynthesis at the log phase, (2) enhancing metabolic flux to TAG biosynthesis at the stationary phase, and (3) suppressing TAG hydrolysis for membrane lipid biosynthesis on growth resumption to maintain accumulated TAG (Figure 2A).

For point 1, to suppress membrane lipid biosynthesis at the log phase, we blocked phosphatidylcholine (PC) biosynthesis by knocking out OPI3, which encodes an enzyme catalyzing the final step of PC biosynthesis (Figure 1). This is because the yeast mutant Δ*opi3* is viable with abolished PC (McGraw and Henry, 1989), whereas mutation in many other rate-limiting enzymes for phospholipid biosynthesis causes lethal effects on growth (Henry et al., 2012). For example, knocking out CDP-diacylglycerol synthase (CDS), which converts PA into CDP-DAG, causes a lethal effect (Shen et al., 1996). A significant metabolic switch is expected to be in favor of TAG production in Δ*opi3* because PC is a primary phospholipid of cellular membranes in *S. cerevisiae*. Indeed, the TAG levels in the stationary phase of Δ*opi3* cells were 4.8-fold higher than that of the wild type based on a dry cell weight (Figure 2B), which agrees with previous study (Fei et al., 2011). We next analyzed the fatty acid composition of TAG, which determines their quality. The Δ*opi3* cells showed an increased composition of palmitoleic acid (16:1) as compared with the wild type (Figure 2C). Therefore, partial blockage of primary membrane phospholipid biosynthesis by knocking out *OPI3* increases TAG with enriched mono-unsaturated fatty acid, giving better quality for biodiesel use.

For point 2, to enhance metabolic flux to TAG biosynthesis at the stationary phase, we first overexpressed *PAH1* (OE-PAH1) to stimulate PA to DAG conversion (Figure 1). The result showed that overexpression of *PAH1* in wild-type cells increased TAG levels by 3.9-fold as compared with the wild type (Figure 2B). The fatty acid composition was not altered significantly by overexpressing *PAH1* (Figure 2C). Since previous report showed that the Δ*pah1* mutant has reduced TAG levels (Adeyo et al., 2011; Fakas et al., 2011b), expression level of *PAH1* may have dose-dependent effect on TAG accumulation.

For point 3, to suppress TAG hydrolysis on growth resumption, we knocked out *DGK1* because previous study demonstrated that DGK1 is required for phospholipid synthesis during growth resumption from stationary phase (Fakas et al., 2011a). In agreement with this observation, TAG content in the Δ*dgk1* cells was increased 1.6-fold as compared with the wild type (Figure 2B), with no remarkable alteration in fatty acid composition (Figure 2C). Thus, suppressing membrane phospholipid biosynthesis, inducing DAG production from PA, and inhibiting TAG mobilization all increased TAG accumulation to a significant extent.

Next, we combined these three genetic manipulations to construct a strain that overexpresses *PAH1* in the Δ*dgk1*Δ*opi3* (Δ*dgk1*Δ*opi3* OE-PAH1). The TAG level was increased 9.7-fold in this strain as compared with the wild type (Figures 2A,B). The fatty acid composition of accumulated TAG was similar to that of Δ*opi3* (Figure 2C), indicating that neither overexpression of *PAH1* nor knockout of *DGK1* altered the fatty acid composition of TAG. Thus, by altering the expression of 3 genes, *OPI3, DGK1*, and *PAH1*, TAG production was increased by 9.7-fold as compared to the wild type in *S. cerevisiae*.

Here, to further increase TAG contents, we overexpressed *CrDGTT2* in the Δ*dgk1*Δ*opi3* OE-PAH1. The resulting TAG levels in Δ*dgk1*Δ*opi3* OE-PAH1 OE-CrDGTT2 were further increased more than 7-fold, giving 69-fold increase as compared to the wild type (Figures 2A,B). The fatty acid composition of accumulated TAG was not altered with overexpression of *CrDGTT2* in the Δ*dgk1*Δ*opi3* OE-PAH1 cells (Figure 2C). To confirm the TAG yield of the Δ*dgk1*Δ*opi3* OE-PAH1 OE-CrDGTT2 cells, we independently established three more strains with the same genotype (CHY140, CHY141, and CHY142) by transforming the plasmid to overexpress *CrDGTT2* into the Δ*dgk1*Δ*opi3* OE-PAH1. When maximal TAG level is produced at late stationary phase (60 h), these cells produced 64.8- to 73.2-fold increase (Supplementary Table 2), indicating reproducibly high yield of TAG in this engineered strain. This TAG amount was estimated to be 12.2% of total dry cell biomass (Figure 2D). This corresponds to 166 mg of TAG per liter of liquid broth culture, which produces about 1.35 g/L of dry cell weight. Thus, our metabolic engineering greatly increased TAG contents using a robust algal gene *CrDGTT2*.

### Characterization of the Δ*dgk1*Δ*opi3* OE-PAH1 OE-CrDGTT2 cells

**Figure 3:**
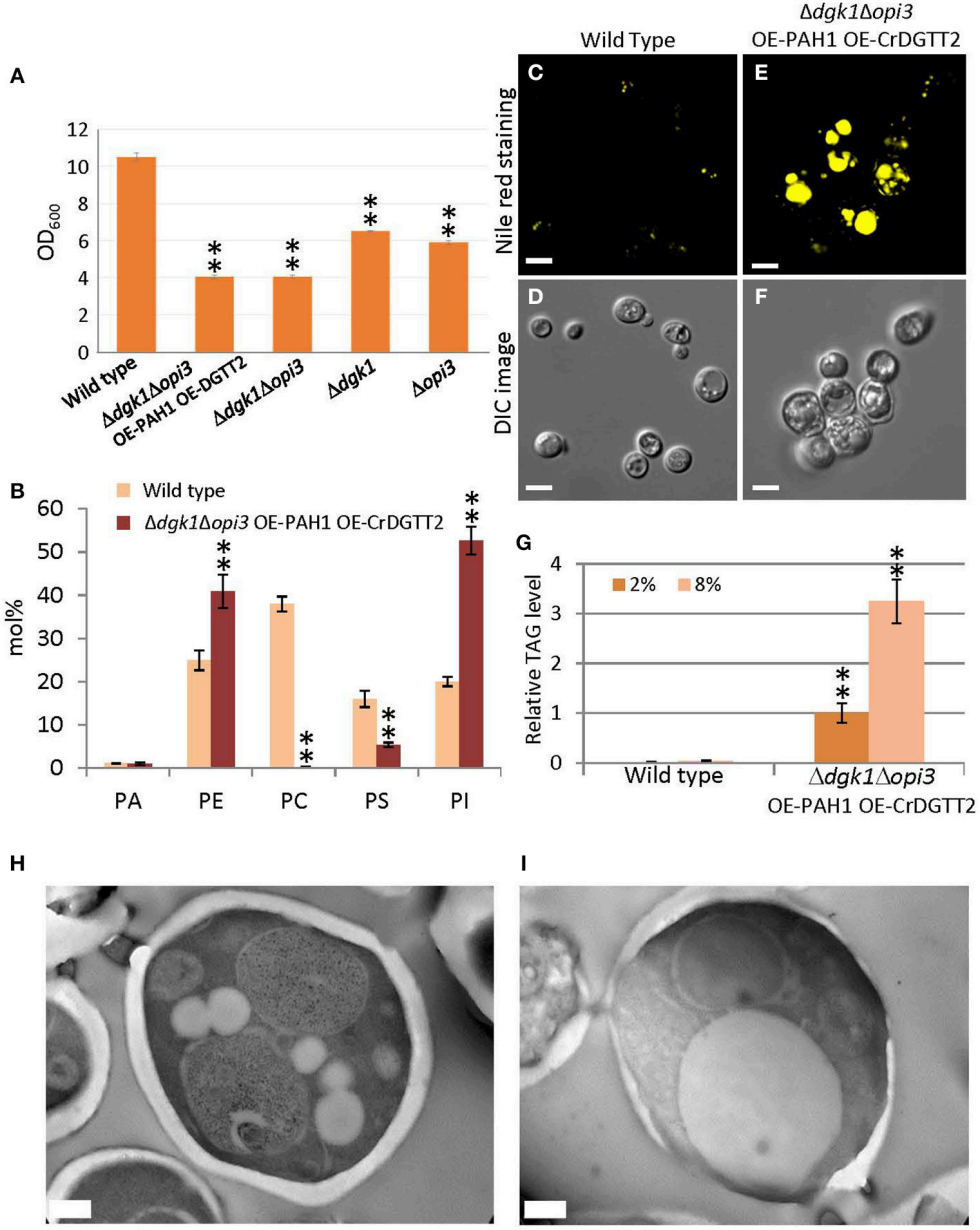
**Phenotypes of the Δ*dgk1*Δ*opi3* OE-PAH1 OE-CrDGTT2 strain. (A)** The OD_600_ value of culture at stationary phase of wild type, Δ*dgk1*Δ*opi3* OE-PAH1 OE-CrDGTT2, Δ*dgk1*Δ*opi3*, Δ*dgk1*, and Δ*opi3*. Fully grown yeast cell cultures were diluted to OD_600_ of 0.1 in 10 ml synthetic complete media at 30°C, and OD_600_ was measured after 72 h of incubation, at which culture reached a plateau. Data are mean ± SD from three biological replicates. **(B)** Membrane lipid composition (mol%) of the Δ*dgk1*Δ*opi3* OE-PAH1 OE-CrDGTT2 strain. Wild type, yellow bars; the Δ*dgk1*Δ*opi3* OE-PAH1 OE-CrDGTT2 strain, brown bars. See Supplementary Table 3 for numerical values. **(C–F)** The Δ*dgk1*Δ*opi3* OE-PAH1 OE-CrDGTT2 strain at the beginning of stationary phase by Nile red staining **(E)** and Normarski differential interference contrast (DIC) microscopy image **(F)** compared with the wild type **(C,D)**. **(G)** Fold increase of TAG levels in response to glucose boost from 2 to 8% at the beginning of stationary phase. TAG level of Δ*dgk1*Δ*opi3* OE-PAH1 OE-CrDGTT2 strain under 2% glucose condition was set as 1. See Supplementary Table 4 for numerical values. **(H,I)** Transmission electron microscope images of the ultrastructure of wild type **(H)** and the Δ*dgk1*Δ*opi3* OE-PAH1 OE-CrDGTT2 **(I)** cells in response to glucose boost from 2 to 8% at the end of log phase. Data in **(A,B)** and **(G)** are mean ± SD from three biological replicates. Asterisks indicate statistical significance by Student's *t*-test (^**^P < 0.01). See Figure 1 for abbreviations. Bars in **(C–F)** are 5 μm and **(H–I)** are 0.5 μm.

We found that the Δ*dgk1*Δ*opi3* OE-PAH1 OE-CrDGTT2 cells showed reduced growth, because culture of the Δ*dgk1*Δ*opi3* OE-PAH1 OE-CrDGTT2 reached a plateau at OD_600_ of 4 while that of the wild type reached a plateau at OD_600_ of 10 in synthetic complete media lacking Ura and Leu (Figure 3A; see Supplementary Figure 1 for the growth curve of the strains). This growth retardation may be due to suppressed membrane lipid biosynthesis rather than massive accumulation of TAG, because the value of OD_600_ was comparable to that of the Δ*dgk1*Δ*opi3* cells (Figure 3A). Indeed, composition of membrane phospholipids was greatly altered as compared to the wild type (Figure 3B; Supplementary Table 3). We also measured level of free fatty acid; wild type cells had 5.8 ± 0.7 μg/mg dry cell weight whereas the Δ*dgk1*Δ*opi3* OE-PAH1 OE-CrDGTT2 cells had 38.2 ± 9.6 μg/mg dry cell weight. Nile red staining for lipid droplets was greater in the Δ*dgk1*Δ*opi3* OE-PAH1 OE-CrDGTT2 cells than wild type cells (Figures 3C,E), which supports massive production of TAG (Figure 2; Supplementary Table 2). The cell size of Δ*dgk1*Δ*opi3* OE-PAH1 OE-CrDGTT2 with TAG accumulation at the stationary phase was larger than wild-type cells (Figures 3D,F). To test whether the Δ*dgk1*Δ*opi3* OE-PAH1 OE-CrDGTT2 cells are still capable of accumulating TAG, we shifted glucose concentration of culture media from 2 to 8% at the end of log phase to trigger extra carbon uptake and fixation to carbon-containing metabolite reserves, including TAG (Kamisaka et al., 2013). As compared with the normal 2% glucose condition, this “sugar boost” further increased TAG level by 3.2-fold (Figure 3G; Supplementary Table 4). These data suggest that the Δ*dgk1*Δ*opi3* OE-PAH1 OE-CrDGTT2 cells have capacity to accumulate extra TAG in response to external glucose supply. We observed the ultrastructure of the Δ*dgk1*Δ*opi3* OE-PAH1 OE-CrDGTT2 cells accumulating TAG under the 8% glucose condition. Compared to the wild type (Figure 3H), the Δ*dgk1*Δ*opi3* OE-PAH1 OE-CrDGTT2 produced super-sized lipid droplets that occupied the primary space in the intracellular compartment (Figure 3I).

### Sustainable TAG production in the Δ*dgk1*Δ*opi3* OE-PAH1 OE-CrDGTT2 cells

A major challenge in TAG metabolic engineering has been how to maintain accumulated TAG with continuous cell growth. This is because TAG accumulation occurs during the stationary phase in wild-type cells, when cell numbers are no longer increased. However, on growth resumption from the stationary phase, accumulated TAG is mostly hydrolyzed to DAG (TAG mobilization) as a substrate to produce phospholipids which are required for rapid growth of cellular membranes (Athenstaedt and Daum, 2005; Kurat et al., 2009).

**Figure 4:**
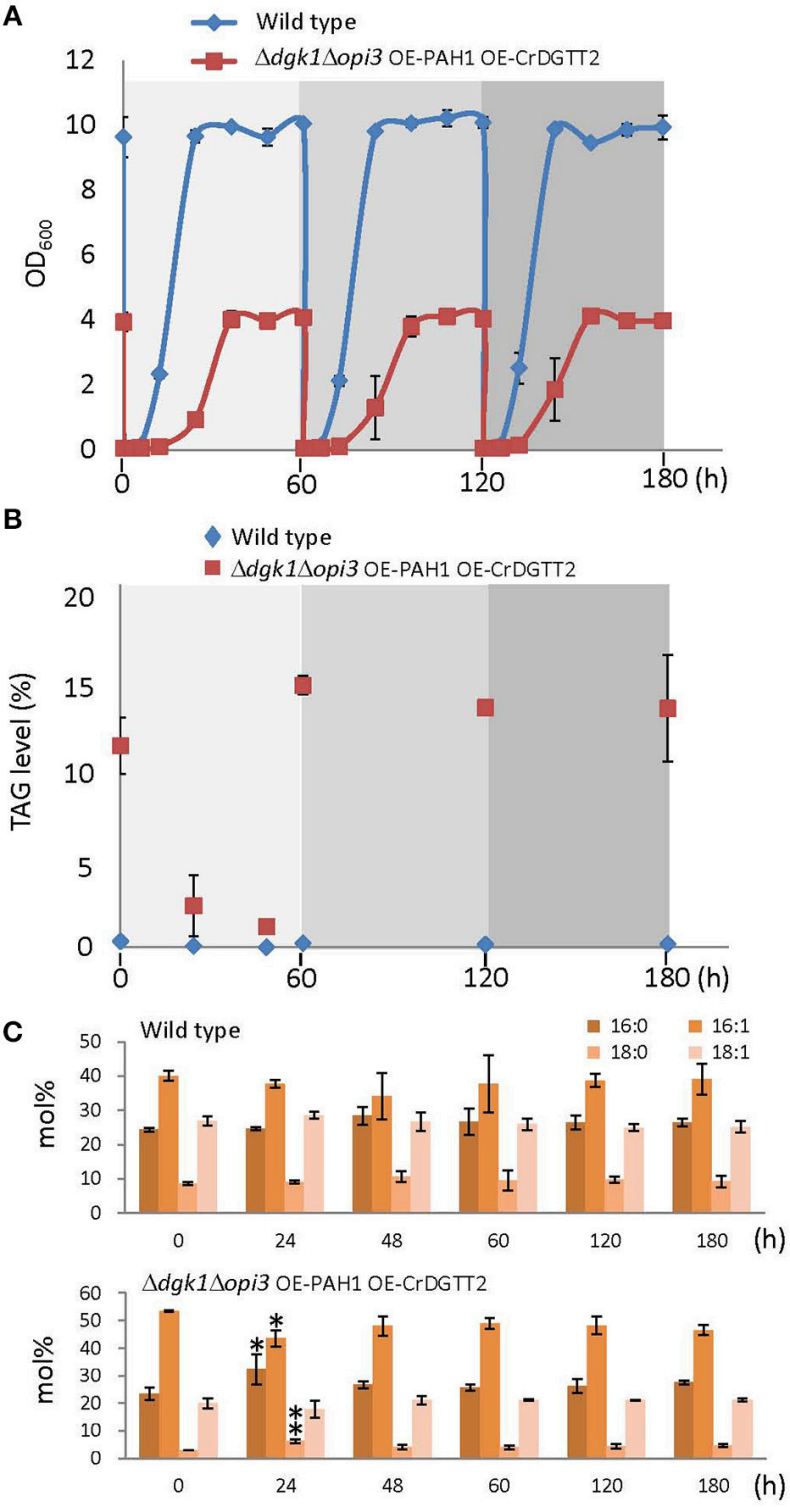
**Sustainable TAG production in the Δ*dgk1*Δ*opi3* OE-PAH1 OE-CrDGTT2 strain. (A)** Growth profile of the Δ*dgk1*Δ*opi3* OE-PAH1 OE-CrDGTT2 strain with repeated resumption of growth. One hundred milliliter of cells at full growth was diluted to OD_600_ of 0.05 at 0, 60, and 120 h. **(B,C)** TAG levels **(B)** and fatty acid composition **(C)** of wild type and the Δ*dgk1*Δ*opi3* OE-PAH1 OE-CrDGTT2 strain with repeated resumption of growth. Data in **(A–C)** are mean ± SD from three biological replicates. Asterisks indicate statistical significance by Student's *t*-test (^*^P < 0.05; ^**^P < 0.01). See Supplementary Tables 5, 6 for numerical values.

To demonstrate whether sustainable TAG production is possible with the Δ*dgk1*Δ*opi3* OE-PAH1 OE-CrDGTT2 cells, quantity (Figure 4B) and quality (Figure 4C) of TAG were profiled along with the cell growth (Figure 4A) through three consecutive repeats of growth resumption. Upon growth resumption from the stationary phase, the growth rate was slower in the Δ*dgk1*Δ*opi3* OE-PAH1 OE-CrDGTT2 than the wild type (Figure 4A); the Δ*dgk1*Δ*opi3* OE-PAH1 OE-CrDGTT2 cells reached stationary phase at 48 h after growth resumption with OD_600_ of 4, whereas wild type cells reached stationary phase at 36 h with OD_600_ of 10. At 60 h, these cultures were diluted again to resume the growth, which reproduced similar growth profile through the following two repeats of growth resumption. TAG level of the Δ*dgk1*Δ*opi3* OE-PAH1 OE-CrDGTT2 cells in full growth at 60 h was 13.2 to 14.5% of dry cell weight (Figure 4B; Supplementary Table 5). Twenty-four hours after growth resumption, TAG level was reduced transiently but was recovered to the initial levels by 60 h. Repeated dilution reproduced the same extent of TAG levels after 60 h for two more times (Figure 4B; Supplementary Table 5). Moreover, fatty acid composition of TAG was maintained throughout the different time points (Figure 4C, Supplementary Table 6), indicating stable quality of TAG. Thus, the Δ*dgk1*Δ*opi3* OE-PAH1 OE-CrDGTT2 cells could grow with reduced degradation of accumulated TAG, which favored sustainable TAG production.

## Discussion

Our metabolic engineering to overexpress an algal gene in genetically manipulated yeast *S. cerevisiae* resulted in about 70-fold increase in TAG level, which is estimated to be 12.2% of dry cell weight (Figure 2D). This yield is superior to recently achieved TAG engineering in *S. cerevisiae*. For example, overexpression of a glycerol kinase and DAG acyltransferases achieved 8.2% of TAG with 2% glycerol as carbon source (Yu et al., 2013). In addition, overexpression of fatty acid biosynthesis genes produced 17% of total lipid fraction including TAG and other lipid classes (Runguphan and Keasling, 2014); because *S. cerevisiae* produces similar amounts of TAG and sterylesters as major lipid content (Beopoulos et al., 2009), our metabolic engineering may produce higher TAG contents. Moreover, our engineered *S. cerevisiae* strain diluted from full growth resumed growth with TAG accumulated (Figure 4). This sustainable TAG production may be an intriguing feature of this engineered strain from the viewpoint of metabolic engineering, although it may have little advantage in real industrial application since dilution requires cost of new medium. Oily yeasts such as *Lipomyces starkeyi, Rhodosporidium toruloides, Rhodotorula glutinis*, and *Yarrowia lipolytica* can produce up to 20% lipids per dry biomass (Runguphan and Keasling, 2014). For oleaginous algae, lipid levels commonly range from 20 to 50% per dry biomass, exceeding the usual yield of oleaginous yeasts (Beopoulos et al., 2009). Although levels of TAG accumulation in the Δ*dgk1*Δ*opi3* OE-PAH1 OE-CrDGTT2 cells were not superior to these oleaginous microorganisms, several advantages of using *S. cerevisiae* over other yeasts or microalgae have been pointed, such as genetic tractability, commercially available whole-genome deletion strain collection or a proven track record in different industrial applications (Tang et al., 2013). Our strategy of metabolic engineering could be applicable to these oleaginous microorganisms to further improve their lipid yields. Molecular engineering of eukaryotic algae still awaits technical developments despite that engineered algae may contribute to the carbon neutrality owing to photosynthesis. Therefore, our TAG engineering using CrDGTT2 provides a new strategy to enhance oil production in *S. cerevisiae*. Since algal species are highly diverse, a robust enzyme similar to CrDGTT2 may be found in other oleaginous algal species. Since CrDGTT2 showed much higher expression level than any other DGTT isoforms in *S. cerevisiae* even though they were all expressed by the same promoter system, it is possible that the robustness of CrDGTT2 may be due to higher stability of mRNA in a heterologous system (Hung et al., 2013). Using algal gene resources to engineer established model microorganism can contribute to develop an innovative approach in the field of synthetic biology and has potential to produce TAG or other value-added oils for industrial demand.

In conclusion, we employed an algal gene resource, *CrDGTT2*, to reconstitute enhanced and sustainable TAG production in a yeast *S. cerevisiae* by metabolic engineering.

## Author contributions

YN conceived research; KK and YN designed experiments; CH performed experiments and analyzed data; KK provided technical assistance to CH, KK, and YN wrote the manuscript; all author commented on the manuscript and approved the contents.

### Conflict of interest statement

The authors declare that the research was conducted in the absence of any commercial or financial relationships that could be construed as a potential conflict of interest.

## Abbreviations

CDP-DAG: cytidine diphosphate diacylglycerol
CL: cardiolipin
DAG: *sn*-1,2-diacylglycerol
G3P: glycerol 3-phosphate
LPA: lysophosphatidic acid
PA: phosphatidic acid
PC: phosphatidylcholine
PE: phosphatidylethanolamine
PG: phosphatidylglycerol
PGP: phosphatidylglycerophosphate
PI: phosphatidylinositol
PS: phosphatidylserine
TAG: triacylglycerol.
