# Supplementary material for: In vivo Reconstitution of Algal Triacylglycerol Production in Saccharomyces cerevisiae
Source: Front Microbiol. 2016 Feb 15;7:70. doi: 10.3389/fmicb.2016.00070 (PMC4753380; doi:10.3389/fmicb.2016.00070)
Supplement: Supplementary file 1 [file Table1.PDF]

Supplementary Tables

Supplementary Table 1 List of oligonucleotide primers used in this study.

Name	Sequences (5’ to 3’)
CH430	CAATTGAAGACAACAAGAATAGCGCAAGTCAAGCGACTCTTGGC CTCCTCTAGTACACT
CH431	CTTCTAACATTATAGAATATATAGAAATAGAGCACCTACATAAGA ACACCTTTGGTGGA
CH374	AATCCCGGGCAAATATCATAAAAAAAGAGAATCT
CH375	GCTTGATCAATTCAAGAAATATCTTGACCGCAGT
CH376	AATTGATCAAGCTTATCGATGATAAGCTGTCAAA
CH377	TTGCCCGGGATTGAAAAAGGAAGAGTATGAGTAT
CH367	CACCATGCAGTACGTAGGCAGAGCTCTTG
CH368	TTAATCTTCGAATTCATCTTCGTCG
