# Supplementary material for: In vivo Reconstitution of Algal Triacylglycerol Production in Saccharomyces cerevisiae
Source: Front Microbiol. 2016 Feb 15;7:70. doi: 10.3389/fmicb.2016.00070 (PMC4753380; doi:10.3389/fmicb.2016.00070)
Supplement: Supplementary file 2 [file Table2.pdf]

Supplementary Table 2 TAG level of three independent strains of Δ*dgk1* Δ*opi3* OE-PAH1 OE-CrDGTT2 (CHY140, CHY141 and CHY142) in comparison with the wild type (CHY044).

Strains	Genotype	TAG level (fold)
CHY044	wild type	1.00 ± 0.19
CHY140	Δ*dgk1* Δ*opi3* OE-PAH1 OE-CrDGTT2	73.2 ± 16
CHY141	Δ*dgk1* Δ*opi3* OE-PAH1 OE-CrDGTT2	73.2 ± 28
CHY142	Δ*dgk1* Δ*opi3* OE-PAH1 OE-CrDGTT2	64.8 ± 16

Data are shown as fold increase compared to the wild type strain and means of three biological replicates with standard deviation.
