# Supplementary material for: In vivo Reconstitution of Algal Triacylglycerol Production in Saccharomyces cerevisiae
Source: Front Microbiol. 2016 Feb 15;7:70. doi: 10.3389/fmicb.2016.00070 (PMC4753380; doi:10.3389/fmicb.2016.00070)
Supplement: Supplementary file 3 [file Table3.pdf]

Supplementary Table 3 Phospholipid composition (mol%) of the Δ*dgk1* Δ*opi3* OE-PAH1 OE-CrDGTT2 shown in Fig. 3B.

	WT	Δ*dgk1* Δ*opi3* OE-PAH1 OE-CrDGTT2
PA	1.10 ± 0.09%	0.98 ± 0.31%
PE	24.94 ± 2.32%	40.89 ± 3.89%
PC	37.96 ± 1.69%	0.20 ± 0.08%
PS	15.98 ± 1.90%	5.34 ± 0.53%
PI	20.01 ± 1.10%	52.60 ± 3.23%
