# Supplementary material for: In vivo Reconstitution of Algal Triacylglycerol Production in Saccharomyces cerevisiae
Source: Front Microbiol. 2016 Feb 15;7:70. doi: 10.3389/fmicb.2016.00070 (PMC4753380; doi:10.3389/fmicb.2016.00070)
Supplement: Supplementary file 4 [file Table4.pdf]

Supplementary Table 4 TAG levels in response to glucose concentration shown in Fig. 3G.

S. cerevisiae strains	2% Glc	8% Glc
WT	0.0149 ± 0.0009	0.0311 ± 0.0131
Δ*dgk1* Δ*opi3* OE-PAH1 OE-CrDGTT2	1.0000 ± 0.1955	3.2453 ± 0.4446
