# Supplementary material for: In vivo Reconstitution of Algal Triacylglycerol Production in Saccharomyces cerevisiae
Source: Front Microbiol. 2016 Feb 15;7:70. doi: 10.3389/fmicb.2016.00070 (PMC4753380; doi:10.3389/fmicb.2016.00070)
Supplement: Supplementary file 5 [file Table5.pdf]

Supplementary Table 5 TAG levels of wild type and the Δ*dgk1* Δ*opi3* OE-PAH1 OE-CrDGTT2 shown in Fig. 4B.

Time	WT	Δ*dgk1* Δ*opi3* OE-PAH1 OE-CrDGTT2
0h	0.418 ± 0.04%	11.13 ± 1.55%
24h	0.068 ± 0.03%	2.003 ± 2.25%
48h	0.014 ± 0.00%	1.153 ± 0.14%
60h	0.254 ± 0.15%	14.46± 0.51%
120h	0.163 ± 0.02%	13.23 ± 0.34%
180h	0.184 ± 0.04%	13.19 ± 2.94%
