# Supplementary material for: In vivo Reconstitution of Algal Triacylglycerol Production in Saccharomyces cerevisiae
Source: Front Microbiol. 2016 Feb 15;7:70. doi: 10.3389/fmicb.2016.00070 (PMC4753380; doi:10.3389/fmicb.2016.00070)
Supplement: Supplementary file 6 [file Table6.pdf]

Supplementary Table 6 Fatty acid composition of wild type and the Δ*dgk1* Δ*opi3* OE-PAH1 OE-CrDGTT2 shown in Fig. 4C.

Strain	FA	0h	24h	48h	60h	120h	180h
**WT**	16:0	24.35 ± 0.55%	24.72 ± 0.46%	28.41 ± 2.61%	26.76 ± 3.79%	26.54 ± 2.00%	26.44 ± 1.17%
	16:1	40.11 ± 1.37%	37.72 ± 1.05%	34.14 ± 6.87%	37.79 ± 8.33%	38.69 ± 1.90%	39.18 ± 4.52%
	18:0	8.65 ± 0.48%	9.03 ± 0.46%	10.75 ± 1.60%	9.54 ± 2.87%	9.83 ± 0.92%	9.21 ± 1.70%
	18:1	26.89 ± 1.44%	28.54 ± 1.05%	26.70 ± 2.66%	25.91 ± 1.67%	24.94 ± 1.02%	25.17 ± 1.65%
**Δ*dgk1* Δ*opi3* OE-PAH1 OE-CrDGTT2**	16:0	23.50 ± 2.22%	32.33 ± 5.40%	26.69 ± 1.20%	25.68 ± 1.07%	26.26 ± 2.41%	27.61 ± 0.71%
	16:1	53.37 ± 0.35%	43.48 ± 3.00%	48.05 ± 3.48%	48.95 ± 1.93%	48.18 ± 3.13%	46.44 ± 1.85%
	18:0	3.09 ± 0.01%	6.30 ± 0.73%	4.17 ± 0.86%	4.04 ± 0.58%	4.34 ± 0.85%	4.70 ± 0.65%
	18:1	20.03 ± 1.87%	17.89 ± 3.13%	21.09 ± 1.41%	21.33 ± 0.28%	21.22 ± 0.13%	21.24 ± 0.48%
