# Supplementary material for: In vivo Reconstitution of Algal Triacylglycerol Production in Saccharomyces cerevisiae
Source: Front Microbiol. 2016 Feb 15;7:70. doi: 10.3389/fmicb.2016.00070 (PMC4753380; doi:10.3389/fmicb.2016.00070)
Supplement: Supplementary file 7 [file Image1.pdf]

In vivo reconstitution of algal triacylglycerol production in *Saccharomyces cerevisiae*

Chun-Hsien Hung[1], Kazue Kanehara[1] and Yuki Nakamura[1,2*]

[1]Institute of Plant and Microbial Biology, Academia Sinica, Taipei 11529, Taiwan
[2]Japan Science and Technology Agency, PRESTO, Saitama, Japan
[*]For correspondence. E-mail nakamura@gate.sinica.edu.tw;
Tel. +886-2-27899590; Fax：+886-2-27827954.

Supplementary Figures

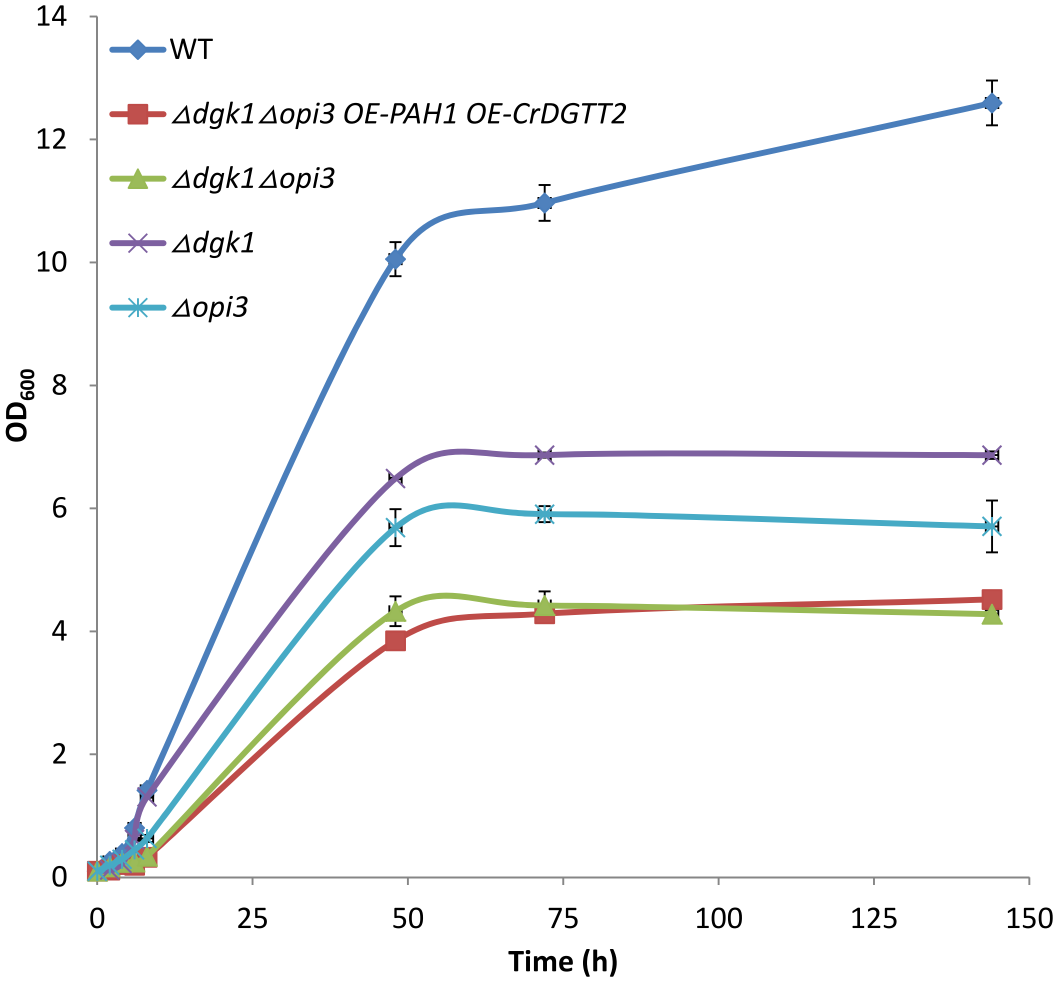

Supplementary Figure 1. Growth profile of wild type, Δ*dgk1*Δ*opi3* OE-PAH1 OE-CrDGTT2, Δ*dgk1*Δ*opi3*, Δ*dgk1* and Δ*opi3*. Fully grown yeast cell cultures were diluted to OD_{600} of 0.1 in 10 ml synthetic complete media at 30°C, and OD_{600} was measured at 0h, 2h, 4h, 6h, 8h, 48h, 72h and 144h. Data are mean±SD from 3 biological replicates.
